# The Human Voice as a Digital Health Solution Leveraging Artificial Intelligence

**DOI:** 10.3390/s25113424

**Published:** 2025-05-29

**Authors:** Pratyusha Muddaloor, Bhavana Baraskar, Hriday Shah, Keerthy Gopalakrishnan, Divyanshi Sood, Prem C. Pasupuleti, Akshay Singh, Dipankar Mitra, Sumedh S. Hoskote, Vivek N. Iyer, Scott A. Helgeson, Shivaram P. Arunachalam

**Affiliations:** 1 Department of Internal Medicine, Lower Bucks Hospital, Bristol, PA 19007, USA; pratyusha205101@gmail.com; 2 Department of Internal Medicine, Mary Washington Healthcare, Fredericksburg, VA 22401, USA; baraskar.bhavana@gmail.com; 3 Department of Medicine, New York Medical College, St. Marys General Hospital & Saint Clare’s Health, Denville, NJ 07834, USA; hshah10@nymc.edu; 4 Department of Internal Medicine, The Wright Center, Scranton, PA 18505, USA; drkeerthygopalakrishnan@gmail.com; 5 UC Health Parkview Medical Center, Pueblo, CO 81003, USA; divyaanshi.sood@uchealth.com; 6 Department of Information Technology, Amity University, Noida 201301, Uttar Pradesh, India; prem.pasupuleti9@gmail.com; 7 College of Computing, Illinois Institute of Technology, Chicago, IL 60616, USA; akshaysinghim@gmail.com; 8 Department of Computer Science & Computer Engineering, University of Wisconsin-La Crosse, La Crosse, WI 54601, USA; dmitra@uwlax.edu; 9 Division of Pulmonary & Critical Care Medicine, Department of Medicine, Mayo Clinic, Rochester, MN 55905, USA; hoskote.sumedh@mayo.edu (S.S.H.); iyer.vivek@mayo.edu (V.N.I.); 10 Department of Critical Care Medicine, Mayo Clinic, Jacksonville, FL 32224, USA; helgeson.scott@mayo.edu; 11 Digital Engineering & Artificial Intelligence Laboratory (DEAL), Department of Critical Care Medicine, Mayo Clinic, Jacksonville, FL 32224, USA; 12 Microwave Engineering and Imaging Laboratory (MEIL), Department of Critical Care Medicine, Mayo Clinic, Jacksonville, FL 32224, USA

**Keywords:** voice, vocal biomarker, voice analysis, vocal features, artificial intelligence

## Abstract

The human voice is an important medium of communication and expression of feelings or thoughts. Disruption in the regulatory systems of the human voice can be analyzed and used as a diagnostic tool, labeling voice as a potential “biomarker”. Conversational artificial intelligence is at the core of voice-powered technologies, enabling intelligent interactions between machines. Due to its richness and availability, voice can be leveraged for predictive analytics and enhanced healthcare insights. Utilizing this idea, we reviewed artificial intelligence (AI) models that have executed vocal analysis and their outcomes. Recordings undergo extraction of useful vocal features to be analyzed by neural networks and machine learning models. Studies reveal machine learning models to be superior to spectral analysis in dynamically combining the huge amount of data of vocal features. Clinical applications of a vocal biomarker exist in neurological diseases such as Parkinson’s, Alzheimer’s, psychological disorders, DM, CHF, CAD, aspiration, GERD, and pulmonary diseases, including COVID-19. The primary ethical challenge when incorporating voice as a diagnostic tool is that of privacy and security. To eliminate this, encryption methods exist to convert patient-identifiable vocal data into a more secure, private nature. Advancements in AI have expanded the capabilities and future potential of voice as a digital health solution.

## 1. Introduction

The human voice is a multifaceted and vital tool for interpersonal communication, facilitating natural and efficient interactions between individuals. It serves as a primary means of exchanging information and allows us to engage in authentic and meaningful social interactions. With a complex array of sounds produced by the vocal cords, the voice carries a wealth of information that enables us to convey emotions or fear, share feelings, and communicate excitement [1]. In the realm of voice analysis, several key parameters are commonly assessed to evaluate voice quality and characteristics. Voice assessment can be assessed subjectively or objectively. Subjective assessment can be performed by grading voice on overall quality (G), roughness (R), breathiness (B), asthenia (A), and strain (S) (the GRBAS scale) [2]. Objective assessment involves the evaluation of multiple acoustic parameters, essentially of four types: glottal features, including information on how the sound is articulated at the vocal cords; tempo-spectral features, comprising acoustic features used in musical information retrieval; formants, including information about the resonance of the vocal tract; and lastly, physical attributes, such as pitch, magnitude, and mean [1,2]. Through voice examination, we gain insights into an individual’s vocal cord characteristics. Voice analysis has found applications in diverse fields, such as speech pathology, forensic investigation, and emotion recognition systems, highlighting its significance in numerous domains [3].

The advent of the Fourth Industrial Revolution has brought forth numerous impactful technologies and artificial intelligence, which have seamlessly integrated into our daily lives and found themselves an especially important place in the medical domain, transforming the scope of medical diagnosis [1]. The introduction of virtual/vocal assistants in smartphones and smart home devices has significantly increased the use of voice-controlled search. In 2019, approximately 31% of smartphone users worldwide utilized voice technology at least once a week, and voice searches accounted for 20% of queries on Google’s mobile app and Android devices [1]. Conversational artificial intelligence (CAI) is at the core of these voice-powered technologies, enabling intelligent interactions between machines such as computers and voice-enabled devices and users through voice and voice user interfaces (VUIs). This convergence of voice technology and artificial intelligence (AI) has made it possible for machines to interact with users in a sophisticated manner. It is now possible to use such technology conjoined with neural networks in analyzing and assessing key vocal parameters, simply at the touch of a smartphone recorder [4]. Adding on, the COVID-19 pandemic has increased the use of video or phone consultations over office visits, benefiting patients who cannot travel or have limited access to medical professionals [3,4].

Speech has been studied extensively in relation to diseases; however, using speech as a diagnostic tool is accompanied by limitations such as the requirement of some degree of language proficiency, accent, language, pathological and physiological influences, and exclusion of those with limited vocal capability. Focusing on voice overcomes these limitations, creating a wider, more inclusive patient population [4,5]. Voice analysis has been employed as a diagnostic tool and a noninvasive biomarker for a range of neuropsychiatric conditions, including Parkinsonism, major depressive disorder (MDD), and diseases like chronic cough associated with gastroesophageal reflux disease (GERD), dyspnea, and even COVID-19 [5,6,7,8,9,10,11]. However, these studies were limited by small sample size, uncertainties associated with sensor measurements, the potential for conscious vocalizing of patients, and unsupervised voice recordings [6,7,8,9,10]. A study conducted using smartphone recording data showed promising outcomes in accurately identifying Parkinson’s disease. This approach has the potential to revolutionize the screening and monitoring of Parkinson’s disease by providing a noninvasive and easily accessible method that utilizes commonly available smartphone technology [12]. Novel applications use phonetic characteristics of voice through machine learning (ML) algorithms to detect cardiac arrest outside of the hospital and to predict pulmonary function in asthma [13,14]. Other studies have explored the association between characteristics of voice signals and coronary artery disease (CAD), adverse outcomes in congestive heart failure (CHF), obstructive sleep apnea (OSA), and the progression of disease in COVID-19 patients [15,16,17,18,19]. Furthermore, analysis of voice has shown promise as a predictor of cognitive decline in vasculature-originated disorders such as diabetes, hypertension, hypercholesterolemia, and heart disease. These studies highlight the potential of voice analysis as a noninvasive and accessible method for detecting and monitoring cardiac and respiratory conditions [18].

There is a need for standardizing corpus collection and establishing a large-scale library of clinically available voice samples. Algorithm optimization, updates, and integration into user-friendly devices such as smartphone applications and connected medical devices are also crucial steps for the future development of vocal biomarkers [14,16,18]. The evolution of neural networks has unlocked numerous potential applications in healthcare. These include the analysis of voice for diagnosis, classification, patient remote monitoring, and improving clinical practices [1]. Expanding the clinical application of voice analysis through artificial intelligence to enable the diagnosis of broader pathologies is looming. It is important to note that no vocal biomarkers have been approved by regulatory agencies like the US Food and Drug Administration or the European Medicines Agency [20]. We review broader medical settings where vocal analysis can prove to be beneficial.

## 2. The Mechanics of Voice

Human voice production is a complex mechanism resulting from fluid structure and acoustic interactions between the anatomy and vibration of vocal cords, activation of laryngeal muscles, and geometric properties of the lungs [21].

### 2.1. Anatomy and Physiology of Voice

The vocal system comprises the vocal folds, vocal tract, lower airways, and the lungs. The lower respiratory tract and lungs supply airflow and pressure modulated by the vibrations of vocal folds, and a voice source is produced. This voice source is then modified by the vocal tract to generate distinct output sounds [21]. The subsequent activation of the five intrinsic laryngeal muscles—namely, the interarytenoid, lateral cricoarytenoid, posterior cricoarytenoid, cricothyroid, and thyroarytenoid—facilitate the adduction/abduction and geometry of the vocal cords to produce sounds [21]. Additionally, the contraction and relaxation of laryngeal muscles regulate the length and tension of vocal cords, leading to alterations in vocal cord tension, which is imperative for voice modulations [21].

A subglottal pressure builds up due to the initiation of airflow caused by lung contractions. When this pressure crosses the required threshold pressure, the vocal cords are pushed apart, allowing the air to escape [22]. As a result of this action, a negative pressure is created in the glottal region. The elastic recoil property of the vocal cords along with the glottal negative pressure leads to the closure of the glottis. The repetition of this cycle causes the vocal cords to vibrate incessantly. Due to these vibrations, the glottal airflow is modulated into a pulsatile flow, which further evolves into a turbulent flow [22]. The vocal sounds thus produced are articulated with the help of the tongue, teeth, lips, and palate to produce consonants and vowels as signaled by the speech areas of the brain cortex: the Broca’s and the Wernicke’s area. These regions facilitate the formation of words and sentences and ensure fluency and coherency of speech [23].

**Figure 1:**
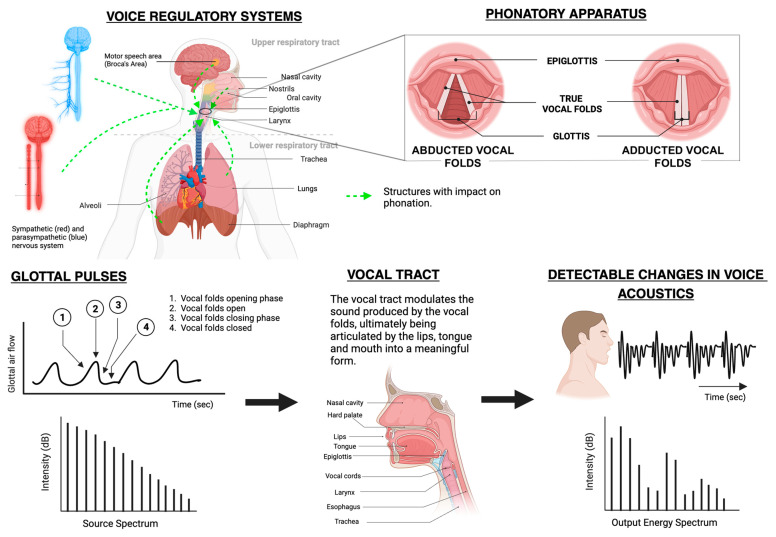
Mechanics of voice production [26].

The degree of approximation of vocal folds, in association with the closure of the glottis and the rate of glottal airflow, determines the quality of voice: breathy, strained, or neutral [24]. Incomplete adduction leads to enhanced turbulent flow through the vocal folds in the presence of high airflow rates, generating a breathy voice. On the contrary, when the vocal cords are in complete approximation, the low airflow rates with peaked subglottal pressure lead to the generation of a strained or pressed voice. The neutral or normal speaking voice is produced due to reduced subglottal pressure in association with reduced airflow rates [24,25]. Figure 1 shows a schematic representation of the mechanics of voice.

### 2.2. Properties of the Human Voice

Vocal parameters commonly used are highlighted in Table 1 [16,27,28,29,30].

## 3. Vocal Analysis Methods

The human voice is a rich source of easily collectable and analyzable high-dimensional data. It has become possible to extract vocal features such as energy, spectrum, and waveform as well as perturbation features [31].

The initial step in creating a digital health solution involves data collection. Voice recordings can include reading a uniform text, counting, spontaneous vocal tasks, and nonverbal phonation such as coughing, breathing, or vowel vocalization [32]. Sustained vowel vocalization has been found superior due to its uniformity; language-independent nature; and the elimination of bias risk from articulatory influences of accent, speaking rates, stress, modulations, and any variable between languages [17,33]. Added benefits of no training prerequisite and stability of analysis are seen [34]. A study of the better vowel for articulation revealed that the vowel /z/ requires precise control over the air gap between the tongue and hard palate with proper positioning and shaping of the lips. The commonly used vowel /a/ demands less precise control, with the jaw open and the tongue at its lowest [17]. The vowel /e/, however, can be achieved even by those with facial palsy or tongue deviations due to the unrounded lips and mid-tongue position [35].

The ideal audio file format is WAV; however, most studies recorded audio files in MP3 format and later used Praat to enhance the file and boost the data quality [26]. Maryn et al. showed the Multi-Dimensional Voice Program (MDVP) to yield higher results than Praat [36]. Raw audio files are preprocessed using methods like standardization, multicollinearity, and dimensional reduction to improve quality before being fed to deep learning (DL) or machine learning (ML) models such as support vector machine (SVM), random forest (RF), K-Nearest Neighbor (KNN), or Naïve Bayes (NB) [32,37,38]. Vocal features can be extracted using Munich Open-Source Media Interpretation by Large Feature-Space Extraction (OpenSMILE), widely studied to extract numerous features such as frame energy, MFCC, loudness, jitter, and shimmer for processing and ML applications [33]. In ML applications, the most crucial features are selected through Least Absolute Shrinkage Selection Operator (LASSO), minimum redundancy maximum relevance, Relief and Local Learning-Base Feature Selection (LLBFS) [38]. Although transfer learning is efficient for small datasets, traditional ML algorithms are powerful for voice analysis for small datasets [3,32,37]. SVM has certain limitations such as limited computation time with large datasets and mathematical complexity [33]. DL models such as artificial neural networks (ANNs) and Convolutional Neural Networks (CNNs) are alternatives to ML for larger datasets due to their success in categorized feature selection and in identifying patterns [37,38].

Vocal biomarkers must undergo authentication of audio quality as well as analytical and clinical validation in order to be defined as a vocal biomarker. After validation, biomarkers can be embedded into digital health solutions such as smartphone apps, chatbots, or voice assistants [32]. Home recordings in natural conditions revealed lower accuracy, sensitivity, and specificity of ML models in detecting vocal abnormalities [37,39]. Carefully constructed datasets in controlled recording environments with efficient preprocessing and fine-tuning of algorithms are fundamental for effective analysis [37].

DL and ML models utilized over the past 10 years in voice analytics for various applications are presented in Table 2, Table 3, Table 4, Table 5 and Table 6 [3,6,7,8,9,10,11,13,15,16,17,19,20,26,33,34,35,40,41,42,43,44,45,46,47,48,49,50,51,52,53,54,55,56].

## 4. Clinical Implications of Voice

Recent research studies have shown the analysis of variations in voice across various medical conditions to diagnose diseases. AI can explore these multidimensional data [57]. Owing to the development of neural networks, we are now able to analyze voice in ways we could not before. Currently, there are no FDA-approved digital voice analytical technologies for clinical purposes, as it is an emerging field and needs more data [58]. Voice biomarkers could help detect certain diseases at early stages for treatment. Various research has been performed using voice analytics to detect neuropsychiatric, cardiac, pulmonary, gastroenterology and endocrine diseases.

### 4.1. Neuropsychiatric Diseases

#### 4.1.1. Parkinson’s Disease

PD is a neurological condition leading to disability, with increasing prevalence compared with other neurological disorders [59]. PD most commonly affects the elderly, with symptoms beginning gradually and worsening over time. With disease progression, patients have memory difficulty, ambulatory issues, speaking and sleep disturbances, and behavioral changes. The standard diagnosis of PD is based on history and clinical symptoms [60]. The literature suggests voice impairment is the earliest sign of motor dysfunction in PD and can manifest in the earliest stages of the disease, worsening with disease severity [9,61]. The correlation between anatomy and physiology in voice impairment in PD has been investigated with various methods such as laryngoscopy, photoglottography, and laryngeal electromyography [61]. Spectral analysis of the voice of PD patients revealed abnormalities such as decreased f0, HNR, and increased jitter and shimmer [9]. Rhonda J et al. examined the voice characteristics of patients with PD according to the disease severity. The study was conducted on 30 early-stage PD and 30 late-stage PD patients. The voices of both groups demonstrated lower mean intensity levels and reduced maximum phonation frequency ranges compared with normal. Patients with late-stage PD had tremors as the main voice feature [62]. Similarly, Harel et al. conducted a case–control study on variability in the frequency of speech of a single individual over 11 years in prodromal PD. The results suggested that changes in variability in speech and acoustic measures can be detected as early as 5 years prior to diagnosis with other methods [63].

The challenges in early diagnosis of PD have inspired the development of ML models using voice data of these patients to detect the disease and its prognosis. Timothy J et al. applied different ML models to classify PD using the mPower Voice dataset and compared it with controls [64]. Using 65,000 10 s voice samples from 6000 people saying /ahh/, it was seen that RF and SVM classifier models were able to differentiate between people with PD 85% of the time with 74% accuracy [64].

Benba et al. tried to differentiate between 20 HCs and 20 patients with PD. Sustained vowels /a/, /o/, and /u/ were collected from subjects, and linear and nonlinear feature extraction were used to obtain the most effective acoustic features for classification. SVM was used for classification, with 87.50% accuracy in differentiating between the two groups [65]. B.E. Sakar et al. compiled voice samples, sound types, sustained vowels, words, and sentences to have a dataset to develop a predicting telemonitoring model for PD. Using time frequency-based feature extraction, the voice samples were grouped into various parameters like frequency, pulse, amplitude, voicing, pitch, and harmonicity. These were fed into SVM and KNN classifiers, which concluded that sustained vowels carried more discriminative information than words and short sentences in PD [66].

Suppa et al. performed an extensive study comparing 30 vocal features between HCs and PD patients, PD patients of early vs. mid-stage disease, PD patients with ON and OFF therapy, and the effect of L-Dopa on voice. They developed an ML model using an SVM classifier and ANN and were successful enough to accurately differentiate the said cohorts. Of the early-stage PD patients, 32% did not display overt voice impairments, which were, however, reported through the ML model, strengthening the prospect of subclinical voice dysfunction recognition. A positive relation between likelihood ratio values computed from the ML model between disease and voice impairment was established, making it a reliable score to dictate voice dysfunction. L-Dopa was seen to have improving outcomes on voice, with an inferior degree of improvement of motor symptoms [9]. These studies suggest that voice can be used as a biomarker to diagnose PD with the help of AI. Further studies with a larger dataset and validation are required to bring this into clinical practice.

#### 4.1.2. Alzheimer’s Disease

Alzheimer’s disease (AD) is the most common cause of dementia worldwide [67]. The National Institute on Aging and the Alzheimer’s Association (NIA–AA) have suggested criteria for the diagnosis of AD according to symptoms and functional impairment. However, definitive diagnosis is based on histopathological examination, which is rarely performed in clinical practice, not to mention being invasive [68]. Early diagnosis of AD is crucial, as it helps patients and caregivers plan for appropriate lifestyle changes to improve quality of life. Various studies have identified the relation between speech changes and AD; however, there is very little information on changes in voice.

With recent advancements in ML, it is possible to explore the acoustic changes in voice for early detection of AD. Cognitive impairment, being an important presentation of AD, can be studied through voice. Mahon et al. studied 10-year cognitive changes from participants of the MIDUS who had completed cognitive testing. The subjects’ age ranged from 42 to 92 years, with an average education of 14.57 years out of 20. The study found increased jitter with higher declines of episodic memory, verbal fluency, and attention switching. Deterioration of episodic memory was also related to lower pulse (*p* = 0.038) and fewer voice breaks (*p* < 0.001). Hence, studying changes in voice could be a predictor of early cognitive impairment, aiding in the diagnosis of AD [27]. Another study conducted an acoustic analysis to identify acoustic parameters in AD and their relation to the anomic impairment. The recorded voice samples were analyzed by the Praat 5.1.4231 program for variables like amplitude disturbance, resonance, and noise disturbance. The study results showed a direct relationship between these acoustic parameters and verbal fluency in AD [69]. ML-based diagnostic models to diagnose AD have been developed, but these models were developed using MRI scan databases [70]. Improved ML models can augment diagnosis at an early stage.

#### 4.1.3. Attention Deficit Hyperactive Disorder (ADHD)

ADHD is a neurodevelopmental condition manifesting in children and characterized by inattention, impulsivity, and hyperactivity [71]. There is evidence that there are neuroanatomical and functional changes that cause these symptoms. Currently, the diagnosis of ADHD is primarily by clinical evaluation based on DSM-5 diagnostic criteria [72]. Recent studies have mentioned the alterations in voice and speech of patients with ADHD. Studies suggest differences in vocal parameters in children with ADHD vs. normal children [73].

Several review studies summarized ML- and DL-based diagnostic methods for ADHD. Most of these studies have ML models developed using MRI, EEG, physiological signals, motion data, and genetic data, but lack studies using voice analytics with ML models, though research in this domain is steadily increasing [74]. Von Polier et al. used an ML-based approach to determine the difference in prosodic voice features between healthy and ADHD patients. Paralinguistic features based on F0 and loudness were analyzed and filtered prior to classification. RF classifications using Tree-Bagger algorithms were able to differentiate ADHD patients from HCs. Increased loudness, hoarseness, and breathiness were seen in ADHD patients compared with HCs. Particularly, those with combined ADHD were louder, had lower F0, more strained voices, and more hoarseness as well as breathiness. This study suggested that voice analysis is promising in diagnosing ADHD [75]. Further research with additional voice features in a larger cohort can improve the classification performance.

#### 4.1.4. Autism Spectrum Disorder

Autism Spectrum Disorder (ASD) is a neurodevelopment disorder associated with behavioral issues, with almost one-third of children intellectually and verbally disabled. Diagnosis is clinically based on communication issues and restrictive and repetitive patterns [76]. Children with ASD mostly develop speech disturbances, but half of these children have distinctive acoustic patterns. They exhibit abnormal voice quality and prosody. A study on pitch variability in the voices of autistic children found that these children had a shallower and less harmonic structure. Abnormal auditory feedback or instability in the mechanisms that control pitch can cause this variability [77]. These atypical vocal characteristics can be used as biomarkers for the early diagnosis of ASD. A systematic review of acoustic patterns in ASD showed that the ML domain can provide promising results for the diagnosis of ASD [77,78].

Asgari et al. developed an ML model that translates prosodic abnormalities into automated, measurable quantities. They developed a harmonic model (HM) using a voice signal and computed a set of quantities related to the harmonic content. They used pitch-related features and employed feature selection to isolate informative prosodic measures. There was a significant association between pitch and loudness with the severity of autism, facilitating the differentiation of autism from HCs [79]. These studies show that ML models using voice patterns can help diagnose autism in younger children with minimal or no language.

#### 4.1.5. Schizophrenia

Schizophrenia (SZ) is a severe neuropsychiatric disorder that occurs in early adulthood, causing emotional, cognitive, and behavioral disturbances with reduced life expectancy [80]. Currently, the diagnosis of SZ is subjective and primarily clinical, with significant symptoms identified by expert physicians. New technologies to reduce the misdiagnosis caused by behavior-based presentations have been attempted [26,81]. Various studies have shown the usage of AI techniques in diagnosing SZ using EEG and MRI findings [80,82]. An overview of AI techniques based on MRI findings has mentioned challenges such as the overlap of MRI findings with those of other neurological disorders and the time-consuming and complicated nature [80].

Atypical voice patterns are seen in SZ, such as increased pauses, distinctive tone, and pitch with varied intensities. These features can be used to develop ML models to diagnose SZ at early stages. A meta-analysis on studies with ML models using acoustic features of SZ found that voice analytics can be promising yet challenging for diagnosis. Most studies used discriminant analysis and SVM to classify patients with SZ. More robust multivariant studies including linguistic aspects such as lexical choices and syntactic and semantic features can help develop better ML models in the future [83]. Compton et al. found that patients with aprosody exhibited reduced variability in pitch, jaw movements, tongue movements, and loudness in voice. The computer program VoiceSauce was used to extract the phonetic linguistic parameter of pitch, and WaveSurfer 1.8.8 was used to extract intensity readings. Praat was used to delineate the vowels from the voice recordings. On comparing these features, results suggested that computational methods can be used to quantify specific negative symptoms in SZ [84]. Voice and prosodic evaluations can help identify the severity of SZ and help diagnose SZ in the early stages.

#### 4.1.6. Mood Disorders

Mood alterations can manifest in facial expressions and through voice; hence, analysis of voice could be of use [85]. Emotional state is primarily controlled by the limbic system; hence, speech mechanisms can be manipulated unknowingly by emotional arousal through the activation of the somatic, sympathetic, and parasympathetic nervous systems [86]. A study group developed a “vitality” index calculating the degree of mental health from voice. This is available as a smartphone/web application (Mind Monitoring System: MIMOSYS, PST Inc., Tokyo, Japan) to monitor mental health and identify mental disorders. The app recognizes changes in F0 to determine the extent of calmness, anger, joy, excitement, and sorrow. Due to diurnal variations, vitality in the morning was found to be more reliable for evaluating mood [85]. Anxiety can be identified as an increase in pitch variability due to trembling of the voice [46].

A study showed glottal acoustic features aided the discrimination accuracy of depressed compared with healthy adolescents [87]. An explanation for this stems from the fact that emotional stress is linked to supraglottal vortices/turbulences as well as psychomotor retardation, leading to increased vocal tract muscle tone and rigidity, evident as monotonous speech and poor articulation [86,87,88]. Differentiation of minor depression from MDD is evident by the lower tone and increased pitch variation in the latter [46].

In an attempt to predict suicidal risk in depression patients, formants and power trends were found to be the favoring vocal features of those at high risk. A pattern of increased formant frequencies and first formant bandwidth, decreased higher formant bandwidths, and glottal spectral slope flattening were associated with depression and suicidality [86,88]. Conversely, most other studies recognized higher energy at upper-frequency bands shifting to lower frequencies post-treatment [86]. Vocal jitter was high and F0 low in those with MDD and imminent suicide risk. The relation of jitter to suicidal risk is due to variations in heart rate and blood pressure, in turn affecting blood flow in the vocal cords, causing erratic vibrations as well as reduced motor unit activation leading to incoordination of the laryngeal muscles. F0 variation was more stressed in patients at the nearest risk of suicide [86].

### 4.2. Pulmonary Disease

There is a close connection between voice and the respiratory cycle, the analysis of which could potentially offer a solution in detecting and diagnosing pulmonary disease. Several studies have shown that vocal biomarkers can be incorporated to diagnose conditions like COPD, asthma, and COVID-19 [89].

#### 4.2.1. Asthma

Asthma is an inflammatory disease of the airways, affecting all ages, that causes constriction of airways and unusual sounds when breathing [90,91]. Peak Flow Meters are used by patients with asthma for daily monitoring in order to record the severity of their condition. Such gadgets are very modest and easy to utilize, yet they need severe cleanliness, which harms the stream sensors simultaneously. It was recognized that pathological voices had higher degrees of jitter and lower levels of HNR [89]. In a study by Dogan et al. that evaluated voice quality in patients with mild-to-moderate asthma using both subjective and objective methods, MPT values were significantly shorter, and average shimmer values higher, for both sexes compared with controls. Female patients with asthma had higher average jitter values compared with sex-matched controls. There was also a significant difference in the VHI and GRB scales between the two groups. The study concluded that in asthmatic patients, MPT, frequency, and amplitude perturbation parameters were impaired, but the vital capacity and duration of illness did not correlate with these findings [92]. The vowel /i/ was a superior choice for speech-based asthma categorization, with a classification accuracy of 80.79% by Yadav et al., whereas wheeze was better for non-speech sounds [90].

#### 4.2.2. COVID-19

Ever since the onset of the pandemic, remote or telemonitoring of COVID-19 patients has been investigated in multiple aspects. The development of remote physiological monitoring of symptoms or recovery of COVID-19 patients would be considered a breakthrough. AI can identify COVID-19 voice changes, which can be uplifted to create smartphone apps or remote monitoring devices as a digital health solution to safely as well as effectively monitor COVID-19 patients [89]. F0 was shown to be beneficial in assessing the normal functioning of the larynx, but shimmer, jitter, and noise in speech signals were symptomatic of pathological instabilities in vocal fold oscillations and inappropriate closure of vocal folds. RF was the most accurate approach for classifying healthy and pathological voices, with an accuracy of roughly 82% in the analysis of three vowels, /a/, /e/, and /o/, for each participant. When only the sound of the vowel /e/ was analyzed, the accuracy increased to 85%. It was also discovered that the vowel /e/ was the most accurate in identifying COVID-19 impacts on voice quality [6]. On the contrary, a study revealed that sustained phoneme features corresponding to vocal tract modulation (MFCC, formants, and VTL) and lung pressure stability (Intensity-SD) were sensitive to COVID-19 infection, and thus, could potentially be used as a COVID-19 biomarker when compared with vocal fold vibration features (jitter, shimmer, pitch, HNR, and NHR). The findings indicate that COVID-19 symptoms that influence laryngeal activity as well as the oral and nasal cavities cause the biggest change in the voice quality of prolonged phonemes. The characteristics retrieved from the vowel /i/ during the first three days following hospital admission were the most successful, with an SVM classification accuracy of 93.5% [17].

Vocal biomarkers are valuable for fatigue monitoring in COVID-19 patients. Voice qualities such as pitch, word duration, and timing of articulated sounds are affected by increased weariness. Consonant sounds that demand a high average airflow require more vocal adjustments due to exhaustion. The authors believe that using voice biomarkers in telemedicine technologies might enhance fatigue monitoring in persons with COVID-19 or long COVID-19 [3].

Researchers found that voice analysis has the potential to increase the accuracy of self-reported symptom-based screening methods for SARS-CoV-2 patients [49]. Some researchers, however, have questioned the relevance of vocal biomarkers for COVID-19 detection, and limitations include patients’ acceptability and readiness for this new technology, as well as health status, which influences adherence to the digital solution [31].

#### 4.2.3. Aspiration

Park et al. used voice-recorded ML algorithms to categorize people with dysphagia who are at risk of tube feeding and post-stroke aspiration pneumonia. This study found that acoustic data acquired using a mobile device can assist in identifying post-stroke individuals who are at high risk of respiratory issues. The XGBoost multimodal model, which incorporated acoustic characteristics, age, weight, and the National Institutes of Health Stroke Scale (NIHSS) score, had an AUC of 0.85 and a sensitivity level of 88.7% in the categorization of patients with tube feeding and a high risk of aspiration. APQ11, shimmer, and RAP were the most significant contributing variables among these metrics [34].

#### 4.2.4. Pulmonary Hypertension

In a study by Sara et al., they evaluated vocal biomarkers and invasively measured indices for pulmonary hypertension. Using voice-processing techniques, a vocal biomarker was developed that retrieved 223 acoustic parameters from 20 s of speech, including MFCC, pitch and formant measures, jitter, shimmer, and loudness. Individuals with greater mean pulmonary arterial pressure (PAP > 35 mmHg) showed substantially higher mean voice biomarker readings than those with lower mean PAP. A one-unit increase in the mean voice biomarker was associated with a high PAP in multivariate logistic regression, implying a relationship between a noninvasive vocal biomarker and an invasively derived hemodynamic index related to PH. These findings might have significant clinical implications for telemedicine and remote monitoring of patients with pulmonary hypertension [19].

### 4.3. Gastrointestinal Diseases

When we consider gastrointestinal diseases, AI networks have shown impressive results in differentiating between benign and malignant lesions, analyzing GI images, and assessing histological diagnosis [93]. We extend the discussion of potential AI applications to voice analytics in gastrointestinal diseases.

In a study by Roldan-Vasco et al., they investigated the application of ML to extract voice attributes from sustained Spanish vowels to explore how swallowing difficulties impact phonation. F0, jitter, shimmer, APQ, PPQ, and energy were among the characteristics recovered. For each feature vector, statistical functions such as mean, standard deviation, skewness, and kurtosis were calculated. These characteristics gave information about the short-term and long-term variability of the voice signal. They were useful in recognizing changes caused by food or liquid residuals in the laryngeal vestibule [31].

In a study by Ayazi et al., voice parameters in normal subjects and GERD patients, as well as the effect of anti-reflux surgery on those parameters, were evaluated. The researchers used electroglottography to measure impedance across the voice cords as participants read a standardized text. Normal participants and GERD patients had their voice frequency, amplitude, and closed-phase ratio computed and compared. When compared with normal persons, patients with GERD exhibited much greater irregularity in both voice frequency and amplitude. After surgery, there was a considerable improvement in both voice frequency and amplitude in GERD patients. In conclusion, GERD impairs voice quality, and anti-reflux surgery reduces voice irregularity in reflux patients [94]. AI implementation would be effective as a noninvasive tool for GERD diagnosis, but it would need a larger store of voice data among GERD patients.

### 4.4. Diabetes Mellitus

DM is a metabolic disorder causing fluctuations in blood glucose levels. These physiological alterations may have implications on an individual’s voice quality. Hamdan et al.’s study, which surveyed 105 patients with T2DM, was one of the first attempts to identify vocal differences between HCs and those with T2DM [95,96].

Individuals with DM demonstrated diminished values in voice parameters including jitter, RAP, shimmer, APQ, smoothed APQ, and NHR when compared with HCs. These conclusions imply that diabetes has a negative impact on voice parameters, and it may assist in the potential identification and differentiation of healthy and pathological voices [97]. Pinyopodjanard et al. showed a significant difference in F0, lower in female diabetic patients compared with controls, through MDVP. This difference remained significant in female diabetic subgroups. However, LR analysis revealed that F0 was not able to predict the presence of diabetes effectively. F0 was found to be a poor predictor of diabetes [56].

Evaluation of vocal variables during episodes of hypoglycemia and hyperglycemia in type 1 diabetes-afflicted individuals was studied. In women, energy (E), amplitude of F0 (AF0), phonation probability (Voiced), formant frequency (F1, F4), residual-to-harmonic ratio (R2H), and harmonic-to-all-energy ratio (Fx3, Fx4) were significantly altered during hypoglycemia, whereas RAP and formant frequency F2 were significantly altered during hyperglycemia when compared with normoglycemia. In men, PERiods (PER), duration of fundamental PERiods (PERTime), Voiced, simple voice quality (SimpleQ), shimmer, APQ, F2, unharmonic-to-harmonics ratio (U2H), subharmonic-to-harmonic ratio (S2H), Fx2, and NHR evidenced differences during hypoglycemia, whereas PERTime, F1, harmonics perturbation quotients (HPQ), U2H, and Fx2 demonstrated significant variation during hyperglycemia (all *p* < 0.05) [98].

A potential relationship between voice characteristics and blood glucose levels has been suggested, but more research with larger datasets is required to confirm these findings and explore the potential application of voice analysis as a noninvasive tool for blood glucose assessment [98,99].

### 4.5. Cardiac Diseases

The influence of the cardiac system on phonation is attributed to the numerous blood arteries existing in the vocal folds. The cardiac cycle causes a physiological variation in F0. During systole, ejection of blood causes swelling of the muscular body of the vocal folds, narrowing the glottis, reducing the glottal closure time, and elevating F0. This physiological process causes cyclical F0 variation during normal phonation. Any condition affecting the heart rate and systolic rate will cause erratic F0 alterations [86].

Although well-established CAD risk markers such as Framingham-based models, ASCVD score, and the Systemic Coronary Risk Evaluation (SCORE) model exist, they fall short by not including a certain subset of patients with preclinical atherosclerosis, sedentary patients, and those with inflammatory disorders. They also consider only traditional risk factors such as hypertension and smoking status, which contribute to less than 70% of CAD cases. Utilizing voice as a supplement to existing markers will allow remote identification of those at risk as well as aid in the management of CAD, reducing the burden on healthcare systems [100]. LR analysis of MFCCs exhibited an association with CAD and voice [101].

Congestive heart failure (CHF) causes vocal fold and pulmonary edema owing to the fluid-retentive nature of the disease, leading to phonation alterations. CHF-related edema can be monitored by body weight; however, this occurs only toward the later stages. The extent of CHF edema required to alter the voice is relatively small compared with the extent required to increase body weight. Hence, collecting glottal, time, and frequency-domain glottal parameters and vocal tract, MFCCs, and acoustic features would provide crucial data. The study revealed that MFCCs had more diagnostic accuracy than glottal features [101]. Reddy et al. documented a more rounded glottal pulse and lower second pressure level (SPL) in CHF patients than in HCs, suggesting imperfect glottal closure leading to inappropriate leakage of air through the glottis, again strengthening the diagnostic value of glottal features [102]. A first-of-its-kind study documented an association between voice and adverse outcomes in CHF, including future hospitalizations and mortality. Acoustic features such as pitch, formant, jitter, shimmer, and loudness were used in creating a linear ML model [15]. The cepstral peak prominence (CPP) vocal parameter was linked to improvement in CHF symptoms post decompensation treatment [101]. There is a need for novel methods of detection of even the minutest decompensation of CHF, and voice analytics might just fill the gap.

### 4.6. Miscellaneous

There is a potential link between endocrinopathies and changes in voice function. The discovery of laryngeal receptors for sex hormones and thyroid hormones shows that alterations in voice may occur as a result of endocrine diseases [2].

**Figure 2:**
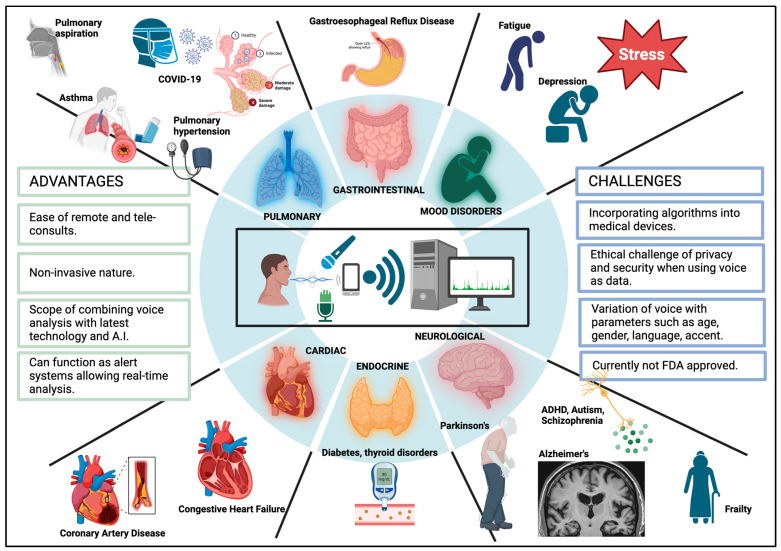
Clinical implications of vocal analytics [26].

A summary of various clinical settings in which vocal analysis can be employed is shown in Figure 2.

## 5. Challenges and Limitations

Using voice as a mode of diagnosis poses certain challenges. To ensure a smooth transition from research of voice technology to clinical practice, certain factors should be considered. Voice being a physiological parameter, it is influenced by language, accent, age, and culture-specific features, which could raise biases [1]. The ideal vocal biomarker integrated to form a digital health solution would be language- and accent-independent. Having said that, narrowing data collection to only vowels or sounds instead of sentences, words, or numbers would eliminate such influences. There is a need to improve natural language processing to understand and analyze vocal recordings [1]. An inevitable concern of recording voice is its identifiable nature, creating issues of privacy and security.

### 5.1. Technical Challenges

Identified challenges with vocal analysis include creating and sharing large databanks with high-quality audio recordings with clinical information and identifying vocal biomarker candidates. Proof-of-concept studies would prove effective. Ensuring audio data synchronization and standardization across studies, in addition to creating more universal accent-, age-, and culture-independent vocal biomarkers, is essential. This can be achieved through replication studies, which can also help to improve algorithm accuracy [1]. This would enable compatibility and transferability, allowing cross-comparisons. Incorporating algorithms into medical devices can be challenging, and qualitative studies with co-design sessions with end users and pilot studies could be beneficial. The embedment of algorithms into already existing IT or telehealth systems is yet to be determined, but it can be aided by randomized controlled trials and real-world evaluation studies [1].

### 5.2. Security, Privacy, and Ethical Challenges

The Health Insurance Portability and Accountability Act (HIPAA) was introduced in 1996 to safeguard patient data under different subgroups that are considered Protected Health Information (PHI) [103]. Voiceprints are information that falls under the umbrella of PHI. Given that they can be used to determine an individual’s identity, demographics, ethnicity, and health status in the context of vocal biomarkers, voice data are regarded as sensitive information [1]. Article 4.1 of the General Data Protection Regulation of the European Union (GDPR EU) states voice as non-anonymous data. It is important to include variable profiles and maintain transparency to minimize systemic biases. Data encryption and random splitting of data for independent processing can be used to address ethical concerns [1].

#### Methods of De-Identification

Audio de-identification or voice de-identification is the process of removing or altering personal information from audio recordings to protect the privacy of users. For the context of our research, audio de-identification aims to preserve the anonymity of patients while still allowing the analysis of their voice data for diagnostic purposes. This process involves several techniques to ensure privacy, enumerated in Table 7 [104,105,106,107,108].

### 5.3. Other Challenges

1. Lack of standardized methods of collecting acoustic data: It is recommended to use an omnidirectional head-mounted microphone distanced 4–10 cm from the lips and at an angle of 45–90° away from the mouth. This allows improved SNRs as well as consistent mouth–microphone distance [109]. As studied by Svec and Granqvist, the microphone should meet the following features, such as having a flat frequency response with a variation of less than 2 dB [110]. The noise level should range from 10 dB lower than the quietest sound to recording the loudest acoustics without clipping [110]. A microphone preamplifier should then augment the captured sound without altering the original signal. This analog signal can be converted to a digital signal through an internal high-quality computer sound card or with an external device, preferably the latter. Characteristics of these converters include a sampling rate of ≥44.1 kHz, minimum resolution of 16 bits, noise level of 10 dB or lower than the quietest sounds, and an adjustable gain to capture the loudest sound with minimal clipping [109,111]. The recommended audio file format is WAV, as it has no compression. The recording environment should be at least 10 dB weaker than the level of the quietest sound. A baseline recording of the background environment should be performed while the subject is quiet for 5 s. Soundproof rooms are preferred [109].
2. Variability and noise: Human voices can exhibit significant variability due to factors like age, gender, speaking styles, accents, or variations in their vocal characteristics. These variations can make it difficult for the model to distinguish between disease-related characteristics and other naturally occurring factors [108].
3. Training set size and quality: The model’s performance relies heavily on the quality and size of the training dataset. Insufficient or unbalanced data can result in biases or reduced accuracy. Obtaining a diverse and adequately sized dataset can be a challenge [108].
4. The dataset should have a good mix of sounds from people with diseases as well as from people who are healthy and do not have any diseases to ensure the model is balanced and not biased toward any one side [1].
5. Optimal design/user experience: There should exist smooth and painless integration of voice analyzing devices in the daily life routine of patients, particularly the elderly, who are not so familiar with technology [4].
6. Reliability and trust: There is the necessity of proving voice as a biomarker in future studies of the accuracy or prognosis assessment capability of these algorithms in diagnosing disease. A need to prove further value above existing technology exists [4].
7. Lack of generalizability and reliability: ML models can often learn unwanted features from datasets, questioning generalizability and reliability. To reduce variability, it is best to use multi-study ML techniques [112].

## 6. Discussion and Conclusions

Medical care has evolved since the pandemic, with video or teleconsultations replacing many hospital visits. Digitization has become the greater focus. Medical devices have long been used to monitor physiological parameters such as blood pressure, oxygen saturation, blood glucose, and cardiac rhythms [31,113]. The human voice is another readily available and collectable physiological parameter containing rich diagnostic clues that could be used for predictive analytics. The notion of using voice as a mode of supplemental diagnosis or as a prognostic indicator is yet to be fully functional. Its noninvasive nature and reduced patient burden, along with the fast and high volume of data collection reducing the health system burden, supports the need to explore more of this domain [1,2,31].

The human voice is an index reflecting characteristics of the vocal cords produced from synchronous interactions of the anatomical, physiological, neurological, respiratory, and cardiovascular systems [2,20,46,114]. Therefore, any alterations in these systems alter certain parameters of the voice, which can be appreciated and analyzed. Artificial intelligence plays a key role in uplifting this approach, creating a new dawn in digital health [1,4,49]. The Fourth Industrial Revolution has presented innovative technologies including voice-powered technologies such as Google Assistant, Alexa, and Siri on smartphones as well as at home, allowing extensive use of voice-controlled search, called CAI. The advent of voice technology, artificial intelligence, and neural networks has opened a way to use voice as a biomarker, creating a potential digital health solution [1,4].

Algorithms already exist for voiceprint recognition to distinguish voiceprint features, although they are limited to legal use and by the FBI [20]. Expanding on this, voice as a biomarker has been studied in various domains, with more focus on neurodegenerative conditions such as Parkinson’s disease, Alzheimer’s, psychiatric conditions, mood disorders, and imminent suicide detection, expanding to pulmonary diseases with increased application in COVID-19 [4,115]. Novel applications have been studied in the identification and prognosis of diabetes, CHF, CAD, GERD, and endocrine disorders. Using phenomes such as vowels makes the vocal biomarker more sensitive due to its language-, accent-, age-, and culture-independent nature. Vocal parameters commonly assessed include the standard amplitude, pitch, pulse, and NSR as well as perturbation features: F0, shimmer, jitter, HNR, and others such as MPT and the S/Z ratio [16,26,27,116]. Glottal, temporal-spectral, and formant features can supplement acoustic findings [46].

Multiple ML and DL models have been studied across various aspects. In relation to PD, KNN showed the highest accuracy for PD male patients, and for female patients, SVM [7]. SVM was also found to have 88% prediction accuracy of fatigue for single vowels and 94% for multi-vowels [20]. OpenSMILE was utilized mainly in MDD studies, but these studies were performed on a small sample size [8,10]. A study performed to evaluate various ML models such as SVM, RF, XGBoost, GBM, adaBoost, Linear, Ridge, elasti net, and LASSO regression to monitor cognitive performance in trauma victims showed XGBoost outperformed the others [45]. A similar study to differentiate between minor and major depression revealed MLP exhibited the best performance (AUC 0.79 for mDE and 0.58 for MDD at 7:3 training set; at 8:2 training set, AUC 0.69 for mDE and 0.67 for MDD) [46]. Various ML models were studied in detecting voice changes in COVID-19 patients from HCs, and the results showed RF (accuracy 82%, sensitivity 94%, specificity 70.59%), Adaboost (accuracy 74%, sensitivity 71%, specificity 76.4%), SVM (accuracy 74%, sensitivity 94%, specificity 52.94%) [6]. XGBoost had higher sensitivity AUC in detecting post-stroke patients at high risk for aspiration [35]. Overall, SVM was found to be best among majority disease spectra for smaller datasets, but for larger datasets, DL models such as ANN and CNN were found to be superior [33,37]. It is to be noted that the ML and DL models have been studied for individual disease analysis. A future exploration would be to analyze a massive dataset of various diseases affecting various systems in the same recording environment using the same model and statistical analysis.

A major challenge with vocal biomarkers is the identifiable nature of voice, potentially violating HIPAA [1]. Encryption methods such as redaction, transformation, cloaking, and noise addition could overcome this [104,105,106,107,108]. The US FDA has not approved vocal biomarkers yet, mainly since this area is so new, and more data are needed. There is also a lack of standardized voice recording or processing methods [1,20]. Novel technological applications are relatively less readily accepted or trusted by people, and hence, they would require convincing evidence of superiority over existing health technologies [4]. There is a lack of generalizability and cross-platform transferability in existing studied models, creating a need for studies with larger cohorts and variable phenotypes [1,109]. Recording from smartphones reduced data accuracy compared with recording with a standardized microphone in a soundproof room with the microphone at a fixed distance [116,117]. Ultimately, a unified corpus collection standard and a large-scale library of clinically available voice samples would be needed. This, followed by algorithm optimization and updates and the incorporation of algorithms into user-friendly devices, should be developed.

### Future Perspectives

Vocal biomarkers can be coupled with smartphone apps, chatbots, smart mirrors, cars, and vocal assistants to monitor symptom resolution, provide information on mental health and quality of life decline, and curate a personalized follow-up post-diagnosis [31,114]. In triage areas of the hospital, vocal screening could be employed [31]. In clinical settings, vocal analysis could be employed for supplementing diagnosis, stratification, and telemedicine/telemonitoring [1]. Vocal biomarkers could be incorporated with alert systems, improving patient safety. Integration with health calls or emergency centers would give real-time analysis of important health-related aspects, supplementing consultations [1,31]. This approach would help describe significant events occurring between two follow-up visits, bridging the existing gap [31].

The development of voice analytic devices as wearable technologies is an area to explore. Constantly improving technology, data-transfer capabilities, 5G networks, and increased usage of smartphones with voice assistants or at-home voice assistants will enable easy collection and processing of vocal data in high definition. It is imperative to adapt natural language processing in voice technologies to understand emotion and empathy in the voice if we are to consider long-term implementation [1]. With future studies incorporating reliable technology with artificial intelligence and larger cohorts of broader phenotypes, voice analysis is a promising digital health solution.

## Figures and Tables

**Table 1:** Commonly used vocal parameters.

Vocal Feature	Definition	Significance
**Basic vocal features** [16,26]		
Amplitude	Extent of vocal cord vibrations	Perceived loudness or softness of voice.
Pitch	Rate of vocal cord vibrations per second	Highness or lowness of sound.
Pulse	Frequency of bursts of air when vocal cords open and close	Unique qualities of a person’s voice.
Noise-to-Signal Ratio (NSR)	Level of desired signal against level of background noise	
**Perturbation features** [16,26,27,28,29]		
Fundamental Frequency (F0)	Total count of sonic waves generated through vocal cords or number of times the glottis opens/closes in a particular time frame	Varies according to gender, ranging between 85–155 Hz in men and 165–255 in women. F0 has a direct relationship with vocal cord length and subglottal pressure.
Shimmer	Variation in amplitude in each glottic cycle or degree of volume instability	Affected by the resistance of the glottis and vocal cord mass lesions. Any value < 1% in children and < 3% in adults is considered pathological.
Jitter, also called Frequency Perturbation	Degree of pitch instability	Highly influenced by uncontrolled vocal fold vibrations. Normal values range from 0.5 to 1.0% for an uninterrupted voice, and higher percentages are suggestive of pathologic voice. As shimmer % and jitter % increase, the voice becomes rougher and quality decreases.
Harmonic Noise Ratio (HNR)	Ratio between vocal fold vibrations (periodic component) and glottal turbulent airflow/noise (non-periodic component) during a voiced segment	Voice is considered more melodious at higher values of HNR and pathological at values lower than 7 dB.
**Additional features** [16]		
Maximum Phonation Time (MPT)	Maximum phonation time of vowel /α:/ after deep inspiration	In glottal pathologies, MPT is decreased.
S/Z Ratio	Calculated by individually making /s/ and /z/ sounds for longest duration after deep inspiration	The S/Z ratio determines the degree of glottic closure and pulmonary functions to help measure adequacy of the laryngeal valve. An increase in S/Z ratio may be seen due to incomplete glottic closure with impaired resonation.

**Table 2:** Voice analytical methods using artificial intelligence in neurological disorders.

Study Purpose	Methodology	Findings of Study	Limitations
**Sajal et al., 2020 [7]** Detection of Parkinson’s disease (PD) in underdeveloped nations by remote data gathering to integrate several symptoms (rest tremor, voice degradation) via smartphones and a cloud-based machine learning system.	Resting tremor data from PD and healthy controls (HCs) recorded with help of three-axis accelerometer sensor built in a smartphone. Samples with sustained phonation of /a/for 10 s were recorded using smartphone at frequencies between 50 Hz and 8 kHz. Jitter, shimmer, HNR, NHR, F0, pitch period entropy (PPE), and recurrence period density entropy (RPDE) features selected using Maximum Relevance Minimum Redundancy (MRMR) algorithm. KNN, SVM, and Bayes classifiers were trained with datasets having combined features of voice and resting tremors for PD analysis.	For male dataset, KNN showed highest accuracy (specificity 89%, sensitivity 100%) for all features, but its accuracy decreased as the features were reduced, and that of SVM and Bayes increased. In female dataset, SVM showed the highest accuracy initially, but as features were decreased, accuracy of Bayes increased with not much difference between SVM and KNN (specificity 97%, sensitivity 99%). For combined male and female data, overall accuracy of all three classifiers was reduced, with KNN showing specificity of 93.7% and sensitivity of 94.6% at 10 features. Tremor data: Accuracy of 98.5% for KNN, 96.8% for SVM, and 91.6% for Bayes in differentiating PD from HC using top eight features. Combined tremor and voice data: combined accuracy for differentiating PD and HC was 99.8%.	The variability in PD severity, intrinsic uncertainty of sensors, and conscious or unconscious suppression of tremors by subjects might lead to inaccuracies in diagnosing PD.
**Suppa et al., 2020 [40]** Diagnosing adductor spasm dysphonia (ASD) and effects of botulinum toxin (BoNT-A) using voice analysis.	A total of 60 ASD patients and 60 age- and gender-matched HCs obtained using two speech tasks: sustained phonation of vowel /e/ for 5 s and reading an Italian sentence. Recorded using H4n Zoom audio recorder at 44.1 kHz. Cepstral analysis was performed using SpeechTool. OpenSmile software used for feature extraction of 6139 features. Weka software fed with SVM algorithm was used for extracted feature analysis. Comparison of cepstral features in patients before and after BoNT-A therapy was performed using paired Student *t* test and among HCs and patients before and after BoNT-A therapy was performed using unpaired Student *t* test.	HC and ASD without BoNT-A: unpaired *t* test showed lower values in patients than in HCs during emission of both vowel and sentence. ASD before and after BoNT-A: Cepstral analysis showed lower values for patients before BoNT-A for emission of vowel and sentence. HC and ASD with BoNT-A: The unpaired *t* test showed lower values in patients than HCs during emission of both vowel and sentence. The SVM classifier using CPS + selected features showed better results than cepstral analysis in all three cases for emission of vowel and sentence.	-
**Suppa et al., 2021 [41]** To assess frequency elements of voice tremor and analyze its response to symptomatic treatment in patients with essential tremors (ETs) with the help of voice recordings.	Voice samples from 58 patients with ET and 74 HCs with sustained phonation of vowel /e/ at normal intensity and pitch for 5 sec recorded in WAV format at 44.1 kHz. ET patients divided into two groups: with voice tremors (ET VT+) and without voice tremors (ET VT−). Spectral analysis of voice samples was performed using Praat. A total of 6139 features were extracted with OpenSMILE. The linear kernel SVM classifier was trained with the 20 most relevant features. Feature selection used Weka software. Unpaired Student *t* test used.	Abnormal oscillatory activity of 2–6 Hz can be seen. Between HC and ET VT+ patients, the SVM classifier showed optimal diagnostic threshold value of 0.88. SVM classifier also showed significant diagnostic performance when ET VT+ and ET VT− groups were compared.	Daily vocal fluctuations were not considered, as voice samples were not serial. Only vocal cord oscillations were considered for vocal tremors, but jaw and neck tremors were not taken into account, which can potentially affect the quality of the voice.
**Tena et al., 2021 [42]** Bulbar involvement detection in amyotrophic lateral sclerosis (ALS) from voice analysis using ML algorithm.	A total of 45 Spanish ALS patients and 18 HCs were asked to provide voice samples with sustained phonation of /a/, /e/, /i/, /o/, /u/ for 3–4 s under medium loudness. Pitch, jitter, shimmer, and HNR were analyzed with Praat. ML classifiers: SVM, NN, LR, LDA evaluated with performance metrics.	At 50% threshold, SVM classifier was 95.8% accurate, followed by NN (94.8% accuracy) and LDA (94.3% accuracy) in identifying ALS with bulbar involvement from HCs. While differentiating ALS without bulbar involvement from HCs, NN showed the highest accuracy of 92.5%.	Small sample size highly influences the significance of the results. Subjects and controls were not age matched.
**Carron et al., 2021 [43]** Methodology to differentiate PD from HC based on smartphone-recorded phonations and to see the effect of uncontrolled environments on these systems.	PD and HCs recruited from two databases: UEX and mPower. The UEX database patients recorded three 5 s /a/ phonations using a model BQ Aquaris V smartphone in a quiet room. MPower database patients recorded /a/ vowel on iPhones or iPods in their own environment. Recordings were trimmed to 1 s using Audacity. A total of 33 features were extracted and preprocessed. Feature selection, hyperparameter optimization, and comparison of performance of six classifiers: LR, RF, SVM, gradient boosting (GB), passive aggressive (PA), Perceptron).	UEX database: PA, Perceptron, SVM, LR: accuracy of >0.9 and greater AUC, processing times of <1 min. PA was superior. Lempel–Ziv complexity (LZ-2), CPP, period density entropy (PDE), fourth and eighth MFCC were highly selected features in the models. MPower database: Lower accuracy, sensitivity, specificity, AUC compared with UEX database findings. GB best among mPower classifiers. Shimmer, MultiFractal Spectrum Width (MFSW), Glottal Quotients, MFCC6 most selected features.	-
**Lin et al., 2022 [34]** Association between frailty (defined through three frailty scales) and voice features in elderly people > 60.	Vowel /a/ for 1 s recorded. Signals digitalized using an A.D. converter. Analyzed with LabVIEW. Four features assessed (average number of ZCR, A1; variations in local peaks and valleys, A2; variations in first and second formant frequencies, A3; spectral energy ratio, A4). Stata software for statistical analysis.	A1 correlated with less likeliness of frailty. A2 was directly proportional to frailty. Gender differences seen: A1 and A3 increase showed increased odds of frailty more strongly in men. In women, stronger association seen with A4. A3 and A4 were a closer match to natural voice. A4 seen to be a good acoustic measure of frailty.	Only 1 s recording taken, missing out on some important vocal features. Use of vowels is not a real mimic of natural language due to varying frequencies and amplitudes.
**Mahon et al., 2022 [27]** Assessment of relation between vocal characteristics and normal 10-year cognitive decline with age from adults in the MIDUS national sample.	Audio clips from patients’ cognitive interviews collected. Brief test of adult cognition by telephone to determine cognitive level. Audio clips filtered to include uninterrupted speech. Six metrics of voice measured (pitch, pulse, voice breaks, jitter, shimmer, amplitude). Longitudinal multilevel modeling (MLM) and lme4 package in R used for analysis.	Age indirectly correlated with pulse and voice breaks and directly associated with jitter and shimmer. Sex associated negatively with jitter, shimmer, and amplitude but positively with pitch, pulse, and voice breaks. Education inversely related to pitch, directly related to pulse, voice breaks, shimmer. Neurological diseases, depression, and chronic conditions negatively associated with pulse, shimmer, and jitter, respectively. Depression positively associated with pitch. Chronic conditions positively related to pulse and voice breaks.	Audio samples obtained during cognitive testing showed significant association with cognition and could suggest dependency. Recordings were of low quality (MP3). The study population was of limited diversity.
**Suppa et al., 2022 [9]** Comparison of PD patients (scored by Hoehn and Yahr scale—H&Y—and UPDRS part III), in early to mid-stages of disease, patients on ON and OFF therapy, PD and controls and effect of L-Dopa on voice.	The study population included 108 HC and 115 PD patients, out of which 57 early-stage had never taken L-Dopa, 58 mid-stage were on chronic L-Dopa therapy—31/58 when OFF and when ON evaluated. Clips of 5 s vowel /e/ and standardized Italian sentence recorded in usual pitch, intensity using high-definition audio recorder H4n Zoom with a Shure WH20 Dynamic Headset Microphone 5 cm from mouth. Preprocessing with OpenSMILE. A total of 6139 vocal features were extracted from each sample. Feature selection using CFS. Graded by relevance by calculating information gain through information gain attribute evaluation (IGAE) algorithm. Top 30 features used in SVM classifier based on linear kernel through MATLAB. Feed forward ANN to calculate degree of voice impairment (likelihood ratio, LR).	SVM classifier: HC and early-stage PD and early- and mid-stage PD—high accuracy for ROC analysis for both vowel and sentence (AUC −0.024, −0.034, respectively). HC and mid-stage PD—greater accurate ROC analysis for vowel vs. sentence (AUC = 0.083). L-Dopa displayed improvement in voice impairment. Mid-stage PD ON and OFF—ROC analysis showed high accuracy for both vowel and sentence (AUC = −0.032). HC and mid-stage ON patients—ROC showed superior classification (AUC = −0.072). Correlation analysis: positive correlation between disease and cognitive challenge vs. voice disability. LR corresponds to the degree of disease and vocal impairment.	Daily physiological voice fluctuations were not taken into consideration. Age differences exist between cohorts.
**Hires et al., 2022 [44]** Detection of PD from voice recordings using CNN algorithm.	Three different datasets: PC-GITA, the Vowels dataset, and the Saarbruecken Voice Database (SVD) with different numbers of study subjects used. PC-GITA and vowel dataset contained sustained phonation of vowels /a/, /e/, /i/, /o/, /u/ at normal pitch; SVD dataset contained sustained phonation of only /a/, /i/, /u/ at normal, rising, high, falling, and low intonations. Short-time Fourier transform (STFT) employed to convert recordings into image data. Spectrogram enhanced using Gaussian blurring. Multiple fine-tuned (MFT) CNN module trained with image datasets. Performance of CNN model was analyzed using ResNet50 and Xception architecture.	The CNN model was able to distinguish between phonation of different vowels in patients with PD and HCs. For vowel /a/, specifically, it showed 99% accuracy, 86.2% sensitivity, 93.3% specificity, and 89.6% AUC.	-

**Table 3:** Voice analytical methods using artificial intelligence in mood disorders.

Study Purpose	Methodology	Findings of Study	Limitations
**Taguchi et al., 2018 [10]** Detection of MDD by analyzing changes in vocal acoustic features.	Groups of 36 patients with MDD and 36 HCs were instructed to read the digits “012-345-6789” followed by speaking the vowels /a/, /u/, /o/ in 30 s, again followed by the digits. Samples were recorded with the help of Google Nexus 7 (TM) tablet at 22.05 kHz. After preprocessing, ‘Feature set of Interspeech 2009 Emotion Challenge’ configuration of OpenSMILE v2.1.0 was used to analyze voice samples. Features assessed were f0, HNR, zero crossing rate, twelve dimensions of Mel-frequency cepstral coefficient (MFCC), and root-mean-square of energy. Statistical analysis performed by Student *t* test.	According to Student *t* test, MFCC-2 was found to be higher in patients with MDD. In the discriminant analysis of MFCC, only MFCC-2 (second generation) was helpful in differentiating between MDD and HCs with sensitivity of 77.8%, specificity 86.1%, and accuracy 81.9%.	Small sample size.
**Wang et al., 2019 [8]** Analysis of vocal differences in HCs versus patients with MDD to diagnose MDD.	A total of 47 patients with MDD and 57 age- and gender-matched HCs were asked to complete four vocal tasks: “Question Answering” (QA), “Text Reading” (TR), “Picture Describing” (PD), and “Video Watching” (VW) to express positive, negative, and neutral emotions. A total of 12 vocal samples were taken for each patient. Feature extraction from the collected voice samples done with openSMILE. Statistical analysis of data was performed using multiple analysis of covariance (MANCOVA).	MANCOVA analysis exhibited that a considerable difference exists in the 12 vocal samples between the patients with MDD and the HCs. Among the analyzed acoustic features, MFCC-5, MFCC-7, and loudness showed difference consistently among the two groups and can potentially be used to diagnose MDD through voice analysis.	The study cannot be generalized due to the small sample size and inclusion of patients with only MDD. The HCs were not matched for education levels with those of MDD patients, which further limited the generalization of the study.
**Schultebraucks et al., 2021 [45]** Development of ML-based voice analysis and other feature areas (facial expressions, movement, speech) to monitor cognitive performance through various domains in trauma victims.	DSM-5 post-traumatic stress disorder (PTSD) victims were interviewed and recorded while answering standardized questions for 3 min. Processing of raw files: Feature selection using RF feature ranking, linear model feature ranking using LR, recursive feature elimination using LR, and stability selection via randomized via LASSO. A total of 247 features were extracted using Parselmouth (Python library). Evaluation of ML models (SVM, RF, XGBoost, GBM, AdaBoost, linear, ridge, elastic net, and LASSO regression). Intensity (dB), formant, pitch variability, amplitude quotient, f0, HNR, glottal to noise excitation ratio calculated.	XGBoost had the best performance using cross-validation. Voice analysis among other feature areas is effective in predicting each cognitive domain.	Difficulty in understanding ML results due to complex nature of models makes it difficult to determine the relation between selected features in the high-dimensional feature space of the developed model.
**Shin et al., 2021 [46]** Diagnosing major and minor depression from vocal biomarkers employing ML.	There were 93 subjects in 3 groups: not depressed (ND), minor depressive episodes (mDEs), and MDD. Objective depression evaluated using the Hamilton Depression Rating Scale (HDRS), subjective depression evaluated using Patient Health Questionnaire-9 (PHQ-9), and anxiety evaluation performed using Beck Anxiety Inventory (BAI). A total of 21 glottal, tempo-spectral, formant, and other physical aspects were extracted. Four ML models: multilayer perceptron (MLP), LR, SVM, and Gaussian Naïve Bayes (GNB) were employed to identify depressive voice changes.	Out of 21 extracted features, 8 showed significant differences. Between the ND and mDE groups, the features spectral centroid, spectral roll-off, standard deviation pitch, voice portion, sq mean pitch, ZCR, and mean magnitude recorded differences. Between mDE and MDD, only standard deviation pitch showed a difference. Among ML models, MLP exhibited the best performance: AUC 0.79 for mDE and 0.58 for MDD at 7:3 training set. At 8:2 training set, AUC 0.69 for mDE and 0.67 for MDD.	The sample size was small because large-scale data collection is time-consuming. The degree of anxiety was not considered while extracting voice features, as anxiety can alter acoustic features in depression. The study could not establish a connection between voice features and depression severity.
**Lee et al., 2021 [47]** Development of a voice-based screening test for depression (VoiSAD) while patients read mood-inducing sentences (MISs).	MDD patients recorded in a quiet room using a Tascam iXZ Microphone fastened to chest while reading out MIS (containing positive, negative, and neutral MISs). Preprocessing with VoiceBox toolkit for Matlab. Feature extraction and analysis with OpenSMILE using audio-visual emotion challenge (AVEC) and Geneva Minimalistic Acoustic Parameter Set (eGeMAPS). A range of 17–43 features selected based on average F score. Classification (AdaBoost classifier).	VoiSAD model: AUC of 0.9 in men and 0.8 in women. Top relevant vocal features in men: Spectral, energy-related features, first MFCC, mean of loudness and audio spectrum, 20% percentile of loudness, root quadratic mean of audio spectrum. Top relevant vocal features in women: Prosody-related features, root quadratic mean of F0, third inter-quartile of F0, mean of f0, 80% percentile of f0, semitone, percentile F0.	Lack of generalizability. Influence of psychotropic drugs on vocal features of drug-dependent patients was not studied. MIS poses a complication of behavioral bias in the patients. Less focus on less-severe depressive diagnosis (dysthymia, adjustment disorder with depression).
**Gao et al., 2022 [20]** Using voice information to detect fatigue in HCs after 36 h of sleep deprivation.	TX650, Sony recorder used to record short text, vowels, phrases from HCs. Denoising and features extraction (f0, energy, zero-crossing-Zcr, HNR, jitter, shimmer, loudness, and 12 MFCCs). Classifiers—LR, linear discriminant analysis (LDA), KNN, classification and regression trees (CART), Naïve Bayes classifier (NB), SVM, MLP to determine fatigue.	Acoustic features for vowel /a/ after 36 h not significantly different than at onset of study. SVM—88% prediction accuracy of fatigue for single vowels, 94% for multi-vowels. CART performed best for speech analysis with accuracy, recall, precision, F1 of 76%, 81%, 76%, 76%, respectively.	Only male acoustics of a certain age group were studied.
**Iyer et al., 2022 [48]** Automatic classification of short speech segments from helpline calls to determine risk of suicide.	Telephone recordings were collected from 281 Suicide Call-Back Service and 000 emergency services and preprocessed to augment signals and reduce noise. Segments of patients’ voice annotated using Audacity. Penalized Lasso regression to identify only vocal predictors (amplitude, frequency, loudness, roughness, spectral slope) with strong association with suicide risk. Generalized additive mixed model and component-wise gradient boosting classification model to predict suicidal risk.	When at imminent suicidal risk, both male and female callers spoke with less signal strength. Increase in spectral slope seen in both sexes as the level of suicidal risk increased. Caller gender was a major moderator. All low-suicide-risk speech frames were correctly classified. AUC of 98.5% was achieved. Only a short segment of audio is required for suicidal risk assessment, and thus, it can be used for triage.	Misjudgment of imminent-risk callers as low-suicide-risk callers causing concern of untimely recognition of those in need of help. Study did not consider members of minority and non-English speaking communities.

**Table 4:** Voice analytical methods using artificial intelligence in pulmonary disorders.

Study Purpose	Methodology	Findings of Study	Limitations
**Sara et al., 2020 [19]** Pulmonary hypertension detection through vocal biomarkers.	Voice samples recorded with three speech tasks for 30 s: R1—reading a prespecified text, R2—describing a positive emotional instance, and R3—describing a negative emotional event. Samples analyzed and converted into vocal biomarkers using Vocalis software in smartphones. Acoustic features: jitter, shimmer, pitch, loudness, and Mel Cepstrum representation were extracted using MFCC. ML techniques were used to calculate biomarkers, and statistical analysis was performed using Student *t* test.	A high mean pulmonary arterial pressure (PAP) was directly proportional to mean values of the voice biomarker (0.74 ± 0.85 vs. 0.40 ± 0.88, *p* = 0.046). Pulmonary capillary wedge pressure (PCWP) and pulmonary vascular resistance (PVR) showed no significant relation to voice biomarkers.	Small sample size, use of only one language (English), and homogeneity of study population limits the generalization of results. The study also fails to provide information on underlying cause of the association between vocal biomarkers and PAP.
**Verde et al., 2021 [6]** Detection of COVID-19 through AI-mediated voice analysis.	Voice samples from 83 HCs and 83 COVID-19 patients were obtained using the coswara database. /a/, /e/, /o/ considered for voice feature extraction of F0, shimmer, jitter, HNR, and MFCC. ML algorithms: Bayes, SVM, SGD, LWL, Adaboost, Bagging, RF, C4.5 Decision Tree trained with these datasets. Weka tool used to perform analysis.	Among the ML models, RF (accuracy: 82%, sensitivity: 94%, specificity: 70.59%), Adaboost (accuracy: 74%, sensitivity: 71%, specificity: 76.4%) SVM (accuracy: 74%, sensitivity: 94%, specificity: 52.94%) showed better performance in detecting voice changes in COVID-19 patients from HCs. Features extracted from vowel ‘e’ able to diagnose COVID-19 patients better than other vowels, with accuracy of 85%.	The voice recordings were unsupervised and contained noises; hence, results cannot be completely validated.
**Maor et al., 2021 [49]** Relationship between a vocal biomarker and COVID-19 infection.	A total of 80 subjects in 2 groups (positive and negative for COVID-19) recorded themselves through Vocalis Health Research mobile app while reading text on the screen. Encoded and 512 features extracted with knowledge transfer using CNN. Two classification models studied (RF, SVM). Statistical analysis using Python and IBM SPSS Statistics software.	Vocal biomarker highest among the COVID-19-positive group, with a one-unit increase in vocal biomarker associated with a rise in the possibility of a positive test of sixfold using a multivariate binary LR. Vocal biomarker detection rate of 69% of asymptomatic COVID-19-positive patients seen.	Lack of generalizability. Study limited to Hebrew language, does not consider possible effects of language on the biomarker. Vocal change seen could also be due to an unrelated respiratory infection.
**Fiorella et al., 2021 [50]** Effect of surgical masks on vocal parameters to determine utilization of surgical masks for safely performing vocal analysis in COVID-19 patients.	HCs recorded sustained /a/ vowel while standing in a silent room 20 cm from a Samson Meteor Mic-USB Studio Condenser Microphone with and without a mask. Praat vocal analysis of pitch (mean, median, min, max), number of pulses and periods, jitter (local, rap, ppq5, ddp), shimmer (apq3, apq5, apq11, dda), HNR.	No significant difference in any parameters of voice with or without a surgical mask.	-
**Elbéji et al., 2022 [3]** AI-based pipeline to create a vocal biomarker for smartphones to monitor fatigue in COVID-19 patients	A total of 1772 voice recordings of 2 types of voice recorded on a smartphone app: one reading a paragraph, one with constant vowel vocalization. Preprocessing using audio clustering (DBSCAN). Feature extraction using transfer learning converted to mel-spectrograms and passed through CNN. Four classification models evaluated to determine fatigue or no fatigue: LR, KNN, SVM, soft voting classifier (VC).	For male iOS and Android users: VC the best model, with AUCs of 85% and 82%, respectively, and accuracy, precision, recall, F1-score of 89% and 84%. For female iOS and Android users: SVM the best, with precision of 79% and 80%, respectively, AUC of 79% and 86%.	Study limited to absence/presence of fatigue only, not severity. Android and iOS users have different microphones, which directly impacts audio quality. Language variations could have resulted in different voice features.
**Higa et al., 2022 [33]** Identifying a voice biomarker for remote monitoring of anosmia and ageusia in COVID-19 patients	Two types of audios: reading an extract and another holding /a/ as long as possible were recorded on a digital app. Raw audio was preprocessed with noise cleaning. Feature extraction using openSMILE. Data divided into 60/20/20 to train, validate, and test ML algorithms (RF, KNN, SVM) to see which classifier is best to detect anosmia or ageusia.	KNN model best for classifying both anosmia and ageusia for both Android and iOS, with AUC of 87% and 80% and precision of 88% and 85%, respectively. Model can effectively distinguish between anosmia and ageusia. Better in detecting the absence of symptoms rather than presence.	Language and accent may alter model performance.
**Pah et al., 2022 [17]** Evaluation of various phenomes and vocal features in differentiating COVID-19 patients with respiratory symptoms.	Sustained phenomes (a/e/i/o/u/m) of COVID-19 patients and HCs recorded in single breath in natural voice using Android phone microphone at 8 kHz and 32-bit resolution daily. One-second segment converted to WAV format for feature extraction using Audacity. Vocal features (jitter, shimmer, SD of pitch, HNR, NHR, formants, VTL, MFCC, intensity) extracted using Praat. MATLAB 2018b used for statistical analysis, Anderson–Darling test used to examine extracted features; Mann–Whitney U test used for comparison of features between both cohorts. SVM to determine effectiveness of vocal features in differentiating COVID-19 from HCs.	The /i/ phenome had the most valuable extractable features to separate HCs from infected, with highest F1 score of 94.3% through SVM classification. Phenome /a/ had the least significant features. Features related to frequency modulation of vocal tracts (MFCC, formants, VTL) and shimmer, intensity more susceptible to vocal changes due to COVID-19. Statistical analysis of voice recordings in first six days of testing positive better in differentiating COVID-19 and HCs.	-
**Park et al., 2022 [35]** To identify post-stroke patients at risk of aspiration and in need of tube feeding using ML models with voice recorded via a mobile.	Patients with dysphagia due to brain lesions recorded phonating /e/ for at least 5 s with iPads. Preprocessing of raw signals using Intel i9 X-series processor and GeForce RTX 3090. Praat software for feature extraction (jitter, shimmer) measured by local, absolute, relative average perturbation (RAP), PPQ5, ddp, local, localdbshimmer, amplitude perturbation quotient (APQ3, APQ5, APQ11), Dda, CPP, and statistical analysis using R statistical software. Evaluation of ML models (LR, decision tree, RF, SVM, GMM, XGBoost).	Groups at higher risk of aspiration had higher standard deviations of amplitude perturbation, f0, frequency, and noise than low-risk patients. XGBoost had higher sensitivity AUC, with RAP, APQ11 shimmer features having the most impact on the model.	Using mobile phones eliminates the use of special equipment, and thus offers safety and efficiency in patients severely ill or at risk of aspiration pneumonia.
**Yaslikaya et al., 2022 [16]** Relationship between voice and obstructive sleep apnea (OSA) and time spent below 90% oxygen saturation (CT90%) during polysomnography.	OSA patients divided into four groups: normal, mild, moderate, and severe according to apnea hypopnea index (AHI). Clips of 5 s recordings of vowels /α:/, /i:/ in isolated audiometry booth using laptop-integrated microphone (SAMSON C01UPRO) and Audacity program. MPT and S/Z ratio calculated. Praat voice analysis. Statistical analysis with SPSS, ANOVA, and Pearson correlation test.	From first to fourth group, MPT was 20.67, 17.06, 15.44, 14.06, respectively, with significant difference between the first and third groups and the fourth group. Mean S/Z ratio was 0.99, 0.90, 0.89, 0.92 from first to fourth group, respectively. Vowel /α:/ No difference in f0. Significant differences in jitter %, shimmer %, HNR between groups. Vowel /i:/ Significant differences in f0, jitter %, shimmer %, HNR between groups. Positive correlation for both vowels between CT90% and shimmer % and negative correlation with HNR. No correlation with f0, jitter %, MPT, S/Z ratio, and CT90%.	Lack of comparison between different acoustic analysis programs. Gender differences not taken into consideration.

**Table 5:** Voice analytical methods using artificial intelligence in cardiac disorders.

Study Purpose	Methodology	Findings of Study	Limitations
**Maor et al., 2018 [51]** Identification of association between CAD and characteristics of voice signals.	Voice data of 101 CAD patients undergoing coronary angiogram and 37 HCs obtained using smartphones. Three data samples were taken from each individual while reading and mentioning a positive and a negative emotional event. Feature extraction performed using MFCCs, and 81 total voice features were extracted. Statistical analysis performed with IBM SPSS version 20.0.	Univariate binary LR model showed association of five voice features with CAD (*p* < 0.05). The multivariate binary regression model revealed that among those five features, only two features showed strong correlation with CAD during emotional events. However, there was no significant difference in these voice features when compared among patients with and without LAD occlusion or in patients who underwent revascularization. Furthermore, these features were not helpful in distinguishing mild, moderate, or severe disease.	This study fails to explain the underlying mechanism of the associations. Small sample size, inclusion of only Caucasian subjects, and usage of only English language. Poor voice quality in the data limits feasibility of this approach.
**Maor et al., 2020 [15]** Evaluating the association between CHF adverse outcomes and vocal biomarkers.	A total of 10,583 subjects were divided into 2 groups: CHF and non-CHF. A 20 s voice sample was obtained from each subject through phone conversation. Vocalis software extracted vocal features: jitter, shimmer, pitch, loudness, and Mel Cepstrum representation. ML model trained with datasets and converted into vocal biomarkers. Statistical analysis performed with ANOVA test.	The univariate Cox regression model exhibited increased death risk of 30% in Q2, 70% in Q3, and 270% in Q4 compared with Q1 as reference (*p* < 0.05 for Q2 and *p* < 0.001 for Q3 and Q4) when the vocal biomarkers were categorized into 4 equal quartiles. The results showed that 1 SD of biomarker was associated with a 48% elevated risk of death (*p* < 0.001) and 25% elevated risk of hospitalization during follow-up (*p* < 0.001) when the biomarker was taken as a continuous variable.	Use of only one language (Russian/Hebrew). This is an observational study; hence, it does not provide concrete data on vocal biomarkers so that it can be used as a stand-alone diagnostic method.
**Rafi et al., 2022 [13]** Detecting cardiac arrest out of the hospital with the help of phonetic characteristics of voice through ML.	Voice samples from 820 calls made to the emergency room by patients whom had out-of-hospital cardiac arrest (OHCA) from 2017 to 2019 were obtained. The f0, intensity, formants, jitter, HNR, shimmer, number of periods, and number of voice breaks features were considered for analysis. ML algorithms: neural network, RF, and LR trained to develop three predictive models.	Among all classifiers, RF showed the best results, with AUC = 74.9, 95% CI = 67.4-82.4.	-

**Table 6:** Voice analytical methods using artificial intelligence to be mentioned.

Study Purpose	Methodology	Findings of Study	Limitations
**Domeracka-Kołodziej et al., 2014 [11]** Assessment of changes in voice quality in patients suffering from GERD-related chronic cough and dysphonia.	A total of 249 GERD patients were divided into 4 groups: men with chronic cough, men with dysphonia, women with chronic cough, women with dysphonia. Assessment of voice quality was performed using MDVP, sonograms, and GRBAS scale.	All groups exhibited vocal changes in objective and subjective analysis. Objective voice analysis using Yanagihara scale revealed lesser degree of hoarseness in GERD patients with cough compared with patients with dysphonia. MDVP analysis in female GERD patients with chronic cough, voice turbulence index (VTI) values less abnormal than those with dysphonia. In male GERD patients with cough, jitter, pitch perturbation quotient (PPQ), relative average perturbation (RAP), and smooth PPQ features were less abnormal.	-
**Ramirez et al., 2018 [52]** To analyze the changes in electroglottography (EGG), acoustic features of voice and Voice Handicap Index (VHI) in patients with laryngopharyngeal reflux (LPR).	Vocal samples of a sustained phoneme (a) phonated for 4 s at natural pitch from 17 patients with LPR and 17 HCs were recorded using a layer spacing microphone. Acoustic features f0, jitter, and shimmer were used for analysis. All the study subjects subsequently underwent EGG using a laryngograph microprocessor EGG-A-100. Vocal samples with sustained phoneme (a) were again recorded and were assessed for open quotient (OQ) and irregularity (%). Statistical analysis was performed using Student *t* test.	Student *t* test demonstrated non-significant difference (*p* = 0.092 for men and *p* = 0.065 in women) in f0 between both groups. Significant difference was found (*p* < 0.05) in jitter and shimmer between both groups. In EGG, the Student *t* test reported higher values of OQ and irregularity percentages in patients with LPR compared with HCs. VHI values were abnormal in LPR, but no significant correlations found between VHI and abnormal acoustic features.	The small sample size and non-homogeneity of study subjects limits the generalization of the study.
**Ruas et al., 2014 [53]** Assessment of voice feature alterations in mucosal leishmaniasis (ML).	Voice recordings from 26 ML patients were analyzed for hoarseness, roughness, and time assessment of the prolonged phonation of vowels A and S/Z using Plantronix-model A-20 microphone. Glottal to Noise Excitation Ratio (GNE), jitter, and shimmer measured. Statistical analysis was performed using Fisher exact test.	Dysphonia was associated the most with pharyngeal lesions (80%, *p* < 0.001), followed by oral cavity (70%, *p* = 0.015), and then the larynx (50%, *p* = 0.004). No difference in voice feature changes according to the position of lesions. Age, gender, alcohol, smoking were not associated with changes in voice.	-
**Mahato et al., 2018 [54]** Assessment of voice quality in schoolteachers pre and post teaching practice.	Voice samples with sustained phonation of ‘i’ were recorded from 60 schoolteachers. Acoustic analysis performed using Doctor Speech (DRS), Tiger Electronics, USA. Acoustic features assessed were F0, jitter and shimmer, MPT, HNR. Statistical analysis performed using SPSS.	The % of shimmer and F0 were increased after teaching practice. HNR and MPT decreased significantly after teaching practice. The values of jitter showed no significant difference.	-
**Pinyopodjanard et al., 2021 [55]** Variations in voice between diabetics (DM) and HCs.	Subjects made 5 s recordings saying the vowel “ah” in one exhalation through microphone 10 cm away from the mouth in a voice laboratory. Parameter extraction: F0, jitter %, shimmer %, APQ, NHR, sAPQ, RAP. Analysis using computerized speech lab model 4500 (CSL) in conjunction with MDVP.	F0 significantly lower in DM than HC. SAPQ significantly higher in DM vs. HC. Upon stratification into genders: No significant difference in features between men and HCs. Female diabetics, women with disease over 10 years, women with neuropathy, women with poor sugar control showed lower F0 than HCs. F0 could not significantly predict DM when other variables were considered.	Absence of laryngeal examination, which could have supplemented findings. Variable baseline characteristics between both cohorts.
**Gölaç et al., 2022 [56]** Comparison of acoustic elements between type 2 DM (T2DM) and HC.	Subject recordings of 91 T2DMs and HCs. Praat software used to evaluate MPT, mean f0, jitter local (Jlocal), jitter absolute (Jabs), shimmer local (Slocal), shimmer decibel (SdB), and HNR.	Only Jabs displayed a statistical difference between both groups. Patients with diabetic neuropathy had statistical differences in MPT and Slocal vs. HCs. T2DMs with voice complaints had differences in Slocal and SdB vs. HCs.	Convincing evidence of relation between DM and voice not established.

**Table 7:** Methods of deidentification of voice.

**Redaction of personal information:** Personal identifiers like name, address, phone number, and other sensitive information are removed from the transcribed text. These identifiers can be recognized through pattern matching, entity recognition, or custom rules.	
**Voice transformation:** To further anonymize the audio, voice transformation techniques can be applied. These methods modify the characteristics of the voice, such as pitch, tone, and speech rate, making it difficult to identify the speaker’s identity. Transformation approaches used to date include the following:	
GMM Mapping	Speaker’s voice is converted to a specific synthetic voice “kal-diphone”.
De-duration Voice Transformation (DurVT)	Changes a person’s voice to sound like another person’s voice, known as a target voice.
Double Voice Transformation (DoubleVT)	Uses two-step transformation: first, it uses DurVT, which produces a synthetic voice “kal-diphone”. Secondly, data from DurVT further undergo transformation using baseline voice transformation technique.
Transterpolated Voice Transformation (Trans VT)	The baseline voice transformation systems essentially perform linear mappings from the features of the source speaker to the features of the target speaker. Transterpolation involves interpolating or extrapolating between the features of the source speaker and the converted features.
**Voice cloaking:** Voice cloaking is a technology designed to alter or disguise someone’s voice to maintain their anonymity or protect their identity. It works by modifying the characteristics of the voice, such as pitch, tone, and timbre, to create a different vocal sound. There are four steps: voice analysis, voice transformation, synthetic voice generation, and playback or real-time processing.	
**Noise addition:** Additional noise can be introduced into the audio to mask any remaining identifiable speech patterns. This can be done by adding background noise or altering the audio signal through filtering or modulation. Noise addition can be additive, multiplicative, or logarithmic multiplicative.	
**Ephemeral Data Storage:** Ephemeral storage is like a temporary storage only when the device is turned on. An example of such temporary data storage arrangement would be the audio-based social media platform “Clubhouse”, which allows users to participate in real-time voice conversations in virtual rooms. The audio content shared in these rooms is not recorded by default. It is deleted after the session ends. A drawback of this approach is that without storing voice data, we might face limitations when it comes to training future ML models.	

## Data Availability

This review was based on publicly available academic literature databases.
